# Exercise alleviates diabetic complications by inhibiting oxidative stress-mediated signaling cascade and mitochondrial metabolic stress in GK diabetic rat tissues

**DOI:** 10.3389/fphys.2022.1052608

**Published:** 2022-12-01

**Authors:** Annie John, Frank Christopher Howarth, Haider Raza

**Affiliations:** ^1^ Department of Biochemistry and Molecular Biology, College of Medicine and Health Sciences, United Arab Emirates University, Al Ain, United Arab Emirates; ^2^ Department of Physiology, College of Medicine and Health Sciences, United Arab Emirates University, Al Ain, United Arab Emirates

**Keywords:** type 2 diabetes, GK rat tissues, exercise, ROS, mitochondria, energy metabolism

## Abstract

Type 2 diabetes, obesity (referred to as “diabesity”), and metabolic syndrome associated with increased insulin resistance and/or decreased insulin sensitivity have been implicated with increased oxidative stress and inflammation, mitochondrial dysfunction, and alterations in energy metabolism. The precise molecular mechanisms of these complications, however, remain to be clarified. Owing to the limitations and off-target side effects of antidiabetic drugs, exercise-induced control of hyperglycemia and increased insulin sensitivity is a preferred strategy to manage “diabesity” associated complications. In this study, we have investigated the effects of moderate exercise (1 h/day, 5 days a week for 60 days) on mitochondrial, metabolic, and oxidative stress-related changes in the liver and kidney of type 2 diabetic Goto-Kakizaki (GK) rats. Our previous study, using the same exercise regimen, demonstrated improved energy metabolism and mitochondrial function in the pancreas of GK diabetic rats. Our current study demonstrates exercise-induced inhibition of ROS production and NADPH oxidase enzyme activity, as well as lipid peroxidation and protein carbonylation in the liver and kidney of GK rats. Interestingly, glutathione (GSH) content and GSH-peroxidase and GSH reductase enzymes as well as superoxide dismutase (SOD) activities were profoundly altered in diabetic rat tissues. Exercise helped in restoring the altered GSH metabolism and antioxidant homeostasis. An increase in cytosolic glycolytic enzyme, hexokinase, and a decrease in mitochondrial Kreb’s cycle enzyme was observed in GK diabetic rat tissues. Exercise helped restore the altered energy metabolism. A significant decrease in the activities of mitochondrial complexes and ATP content was also observed in the GK rats and exercise regulated the activities of the respiratory complexes and improved energy utilization. Activation of cytochrome P450s, CYP 2E1, and CYP 3A4 was observed in the tissues of GK rats, which recovered after exercise. Altered expression of redox-responsive proteins and translocation of transcription factor NFκB-p65, accompanied by activation of AMP-activated protein kinase (AMPK), SIRT-1, Glut-4, and PPAR-γ suggests the induction of antioxidant defense responses and increased energy metabolism in GK diabetic rats after exercise.

## Introduction

Type 2 diabetes, the most common metabolic disorder, is increasing rapidly worldwide and in the Middle East in particular (El-Kebbi et al., 2021). The global diabetes prevalence in 2021 was estimated to be 536.6 million people rising to 783.2 million by 2045, with the prevalence higher in urban than rural areas (Sun et al., 2022). Studies have shown that type 2 diabetes is characterized by chronic hyperglycemia due to a reduction in insulin response, inflammation, metabolic dysregulation, increased formation of free radicals, and decreased antioxidant capacity (Golbidi et al., 2012; Foudi and Legeay, 2021), eventually causing disease complications (Liu et al., 2019). The Goto-Kakizaki (GK) rat, produced originally by selective inbreeding for a hyperglycemic trait, is a widely used model for type 2 diabetes (Guest, 2019). Type 2 diabetes, obesity, and metabolic syndrome (hereafter referred to as “diabesity”) associated with increased insulin resistance and/or decreased insulin sensitivity have also been implicated with mitochondrial dysfunction and alterations in energy metabolism. The precise mechanisms leading to the progression of these complications are still unclear. Owing to the limitations and off-target side effects of antidiabetic drugs, exercise-induced control of hyperglycemia and increased insulin sensitivity is a preferred line of strategy to manage “diabesity” associated complications. Uncontrolled hyperglycemia is a risk factor for diabetes and its complications including cardiovascular disease. Most current anti-diabetic therapies increase insulin levels or enhance insulin action.

Exercise-induced management of type 2 diabetes is the first line of choice to manage hyperglycemia, hyperlipidemia, and other diabetes-associated complications. Exercise has been known to attenuate oxidative stress and improve diabetic complications due to its effects on mitochondrial carbohydrate and lipid metabolism (Qi et al., 2011). Mitochondrial function is the key to metabolic homeostasis in metabolizing nutrients into ATP and is also tightly coupled with redox homeostasis (Tanaka et al., 2020).

Our earlier study on GK rats subjected to moderate exercise training has shown improvement in cardiac muscle contractile dysfunction and relaxation in myocytes, which are relevant to improvement in diabetes-induced myocardial dysfunction (Salem et al., 2013) Our studies have also demonstrated alterations in redox metabolism, oxidative stress, and mitochondrial dysfunction in the tissues of ZDF type 2 diabetic rats, another animal model of type 2 diabetes (Raza et al., 2012; Raza et al., 2013; Raza et al., 2015). Increased oxidative stress and impaired metabolic and mitochondrial functions were also observed in different tissues of STZ-induced type 1 diabetic rats (Raza et al., 2004; Raza et al., 2011). GK diabetic rats subjected to controlled exercise regimens demonstrated that increased oxidative stress due to increased production of reactive oxygen species (ROS), lipid/protein peroxidation, and nitric oxide (NO) production resulted in alterations in glutathione (GSH)-dependent antioxidant defense metabolism which protects the pancreas from diabetes-induced pathological changes (Raza et al., 2016).

Exercise training is recognized as an important non-therapeutic strategy in the management of type 2 diabetes, and studies have shown its effects on mitochondrial metabolic, oxidative, and redox functions in different tissues. Alterations in ROS production, energy, and metabolic homeostasis are dependent on mitochondrial functions. Exercise may perturb metabolic homeostasis and enhance the utilization of nutrients and oxygen which may alter mitochondrial function. However, a clear understanding of the molecular basis for redox and metabolic homeostasis, the precise metabolic targets and the exact mechanism of improved metabolic function after exercise training is still not clear. We, therefore, aimed to investigate the beneficial effects of exercise on metabolic and redox homeostasis in two energy metabolizing and homeostatic tissues, the liver, and kidney, from GK diabetic rats and have compared them with non-diabetic Wistar rats. In addition, we also studied the effects of exercise on metabolic adaptation and energy utilization in these rats in comparison with the sedentary rats.

## Materials and methods

### Chemicals and reagents

Cytochrome c, reduced glutathione (GSH), oxidized glutathione (GSSG), 5,5′-dithio-bis(2-nitrobenzoic acid), cumene hydroperoxide, dimethylnitrosamine (DMNA), erythromycin, dinitrophenylhydrazine (DNPH), lucigenin, glutathione reductase, thiobarbituric acid, NADH, NADPH, coenzyme Q2, antimycin A, sodium succinate, dodecyl maltoside, Hexokinase assay kits, and ATP Bioluminescent cell assay kits were purchased from Sigma-Aldrich Fine Chemicals (St Louis, MO, United States). 2′, 7′- Dichlorofluorescein diacetate (DCFDA) was procured from Molecular Probes (Eugene, OR, United States). Kits for SOD assay were purchased from R & D Systems (Minneapolis, MN, United States) and for glutamate dehydrogenase from Abcam (Cambridge, United Kingdom). Polyclonal antibodies against protein kinase B (Akt), phosphorylated protein kinase B (p-Akt), AMPK, p-AMPK, NFkB-p65, IkB, Nrf2, VDAC, and glyceraldehyde 3-phosphate dehydrogenase (GAPDH) were purchased from Cell Signaling Technology, Inc. (Danvers, MA, United States). Antibodies against cytochrome c were purchased from Santa Cruz Biotechnology Inc. (Santa Cruz, CA, United States) and against Heme Oxygenase-1(HO-1), PPAR-γ SIRT-1, Glut-4, and Aconitase from Abcam (Cambridge, United Kingdom) and for Hsp-70 from Sigma-Aldrich Fine Chemicals (St Louis, MO, United States). Reagents for SDS-PAGE and Western blot analyses were purchased from Gibco BRL (Grand Island, NY, United States) and Bio-Rad Laboratories (Richmond, CA, United States).

### Experimental animals

Eleven-month-old male type 2 diabetic GK rats (*n* = 30) were purchased from Taconic (Germantown, NY, United States). The same number of male control Wistar rats of similar age were procured from the College of Medicine and Health Sciences, Animal House facility of the U.A.E. University, U.A.E. Animals had free access to food and water and were maintained under a standard 12 h light/dark cycle. Animals were constantly monitored during the period of the study. Animal ethical approval was obtained from the Ethic Committee of the College of Medicine and Health Sciences, U.A.E. University, U.A.E.

### Exercise training protocol

The two groups of animals were further subdivided into two groups, with each of the sub-groups containing 15 animals. One subgroup each under GK rats and control Wistar rats respectively received aerobic exercise training whilst the animals in the other sub-group from the two groups remained sedentary. The treadmill training protocol was performed for 1 h daily on an EXER-4 treadmill (Columbus Instruments, Columbus, OH, United States) as described before (Salem et al., 2013). Exercise training was done regularly for 5 days a week for a total of 8 weeks. Each of the training sessions started with a warm-up, during which the belt speed was gradually increased. Over the weeks, the belt speed was gradually increased from 10 m/min to 15 m/min by week 4, after which the belt speed was increased and maintained at 20 m/min for the remaining period of the exercise regimen. Animals were regularly monitored over the 2 months as described before (Salem et al., 2013).

At the end of the training period, the animals were sacrificed, and the liver and kidneys were quickly excised from the animals and stored immediately at −80°C until further analysis (within 1 week). For cellular fractionation, the tissues were homogenized in H-medium (70 mM sucrose, 220 mM mannitol, 2.5 mM HEPES, 2 mM EDTA, 0.1 mM phenylmethylsulfonylfluoride, pH 7.4) at 4°C and the fractions separated by differential centrifugation and the purity of the fractions were checked as described before (Raza et al., 2004; Raza et al., 2011; Raza et al., 2015). The protein concentration of the individual fractions was measured by the Bradford method (Bradford, 1976) as described before (Amiri et al., 2015; John et al., 2021; John et al., 2022).

### Measurement of ROS production, NADPH oxidase (NOX) activity, and lipid and protein oxidation

ROS production was measured in the liver and kidneys of exercised and sedentary control Wistar and type 2 diabetic GK rats using the DCFH-DA fluorescence method to measure the total peroxides as described before (Raza et al., 2004, 2016; John et al., 2021; John et al., 2022). Non-mitochondrial ROS was measured as the NADPH oxidase (NOX) activity by the lucigenin-enhanced chemiluminescence method and the luminescence signals were read immediately using the TD-20/20 luminometer (Turner Designs, Sunnyvale, CA, United States). Microsomal NADPH-dependent lipid peroxidation was measured by the thiobarbituric acid method using malondialdehyde as standard as described earlier (Raza et al., 2004; Raza et al., 2011; Raza et al., 2015; Raza et al., 2016). The effect of oxidative stress on proteins was measured as protein carbonylation using dinitrophenylhydrazine (DNPH) as substrate as described before (Raza et al., 2004; Raza et al., 2011; Raza et al., 2015; Raza et al., 2016).

### Measurement of GSH level, and GSH-peroxidase, GSH reductase, and SOD activities

The enzymatic and non-enzymatic physiological antioxidants play an important role in oxidative stress defense. Alterations in glutathione levels and GSH-redox metabolism are key indicators of increased oxidative stress. Total GSH levels were measured as the conversion of oxidized glutathione to reduced glutathione by glutathione reductase using DTNB (5,5′-dithio-bis (2-nitrobenzoic acid) as substrate. Glutathione reductase and glutathione peroxidase (GSH-Px) activities were measured using GSSG/NADPH and cumene hydroperoxide as substrates respectively as described previously (Raza et al., 2004; Raza et al., 2011; Raza et al., 2015; Raza et al., 2016). SOD activity was measured using the SOD assay kit (R & D Systems, Minneapolis, MN, United States). The percent conversion of NBT to NBT-diformazan was calculated.

### Measurement of microsomal cytochrome P450 2E1 and 3A4 enzymes

Cytochrome P450-dependent monooxygenase enzymes play important roles in maintaining redox homeostasis and detoxification of various drugs. Our previous studies have also shown the activation of cytochrome P450 enzymes in type 1 and type 2 diabetic rat models (Raza et al., 2004; Raza et al., 2015; Raza et al., 2016; John et al., 2022). Using dimethyl nitrosamine (DMNA) and erythromycin as substrates for CYP 450 2E1 and CYP 450 3A4 respectively, we have measured CYP 450 2E1 and CYP 450 3A4 enzyme activities as described earlier.

### Measuring the activities of energy-metabolizing enzymes

The activity of the rate-limiting glycolytic enzyme, hexokinase was measured using the Hexokinase assay kit (Sigma-Aldrich Fine Chemicals, St Louis, MO, United States) as per the manufacturer’s protocol. Briefly, the assay involves the enzymatic conversion of glucose to glucose-6-phosphate coupled with glucose-6-phosphate dehydrogenase, and the NADH thus formed reduces a colorless probe to a colorimetric product, which is then read at 450 nm.

The activity of the mitochondrial enzyme, glutamate dehydrogenase (GDH) was measured using the GDH assay kit (Abcam, Cambridge, United Kingdom). Briefly, the assay consisted of measuring NADH generated by the enzymatic conversion of glutamate, spectrophotometrically at 450 nm.

### Measurement of mitochondrial function and bioenergetics

Activities of mitochondrial respiratory complexes from freshly isolated mitochondria were measured in the liver and kidney of control Wistar and diabetic GK rats, with or without exercise. Activities of NADH-ubiquinone-oxidoreductase (Complex I), succinate-cytochrome c reductase (Complex II/III), and cytochrome c oxidase (Complex IV) were measured by the methods of Birch-Machin and Turnbull (Birch-Machin and Turnbull, 2001) using coenzyme Q2, succinate and reduced cytochrome c as substrates as described before (Raza et al., 2004; Raza et al., 2011; Raza et al., 2015; Raza et al., 2016; John et al., 2022). The total ATP levels in the mitochondrial fractions were measured using the ATP somatic cell assay kit (Sigma-Aldrich Fine Chemicals, St Louis, MO, United States) and the luminescence was read immediately using the TD-20/20 luminometer (Turner Designs, Sunnyvale, CA, United States).

### Assessing protein expression by SDS-PAGE and western blotting

Cellular fractions (mitochondria, cytosol, 30–50 µg protein) from the liver and kidneys of control Wistar and diabetic GK rats were electrophoretically separated by 7.5%–12% SDS-PAGE (Laemmli, 1970) and transferred onto nitrocellulose membrane (Towbin et al., 1992). The membranes were then immunoblotted with stress-sensitive marker proteins, Hsp-70, mitochondrial aconitase and cytochrome c, HO-1 and Nrf2, inflammatory markers, NFκB-p65 and IκB, and cell-signaling regulatory markers, AMPK, p-AMPK, SIRT-1, Akt, p-Akt, Glut-4, and PPAR-γ. The immunoreactive proteins were then visualized using the Sapphire Biomolecular Imager (Azure Biosystems, Dublin, OH, United States) or by exposure to X-ray films. GAPDH and VDAC were used as the respective loading controls for cytosolic and mitochondrial fractions. Image Studio Lite, ver.5.2 (LI-COR Biosciences, Lincoln, NE, United States) was used for the quantitative analysis of the proteins and plotted as ratios of the protein to loading control or phosphorylated protein to total protein.

### Statistical analysis

Values are expressed as mean ± SD of three-five biological replicates. Statistical analysis of the data was done using SPSS software (version 23) using analysis of variance followed by post hoc LSD analysis for multiple comparisons. Statistical significance was set at *p* < 0.05.

## Results

### Exercise-induced alleviation of oxidative stress in GK rat liver and kidney

**FIGURE 1:**
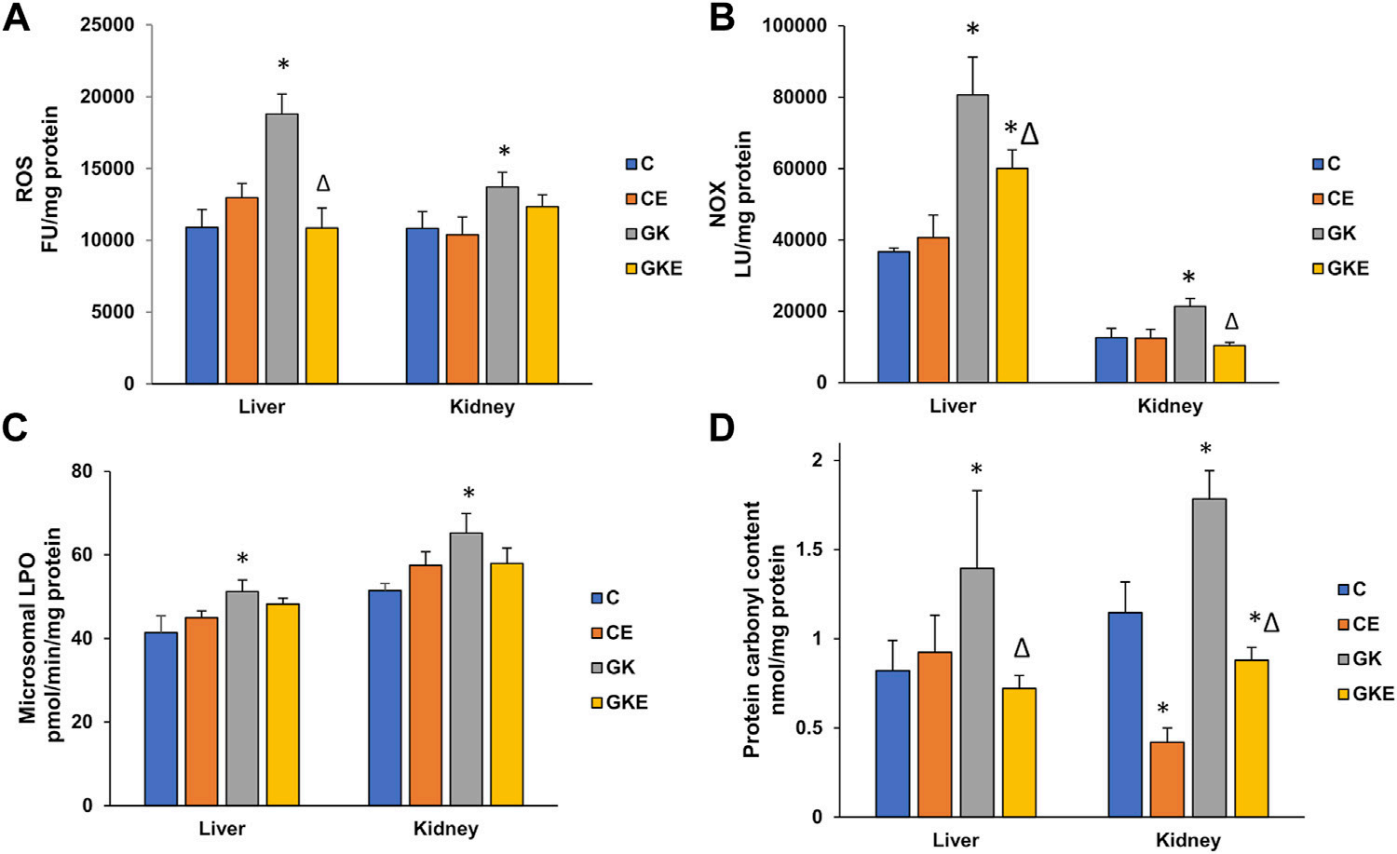
Exercise-induced alleviation in ROS and NOX production, lipid and protein oxidation in the liver and kidney of control Wistar (C) and diabetic GK (GK) and exercise-trained control (CE) and GK (GKE) rats (*n* = 5). At the end of the exercise training for 2 months, the liver and kidney tissues collected from control and GK rats from each of the sub-groups were homogenized and analyzed for ROS production **(A)** measured using 2′, 7′-DCDF fluorescence assay, membrane-bound NOX production **(B)** measured using the lucigenin-based assay as described in the Materials and Methods. Microsomal lipid peroxidation **(C)** was measured by the thiobarbituric acid method using malondialdehyde as standard and protein carbonylation **(D)** was measured using dinitrophenylhydrazine (DNPH) as substrate. Histogram values are mean ± of three independent experiments and statistical significance is shown as asterisks, which was fixed as *p* < 0.05 (* indicates statistical significance compared with control Wistar rat tissues while ∆ indicates statistical significance compared with diabetic GK rat tissues).

To check the beneficial effects of exercise on oxidative stress, ROS production, NOX activity, and lipid and protein oxidation parameters were studied (Figure 1) and compared with exercise-trained control Wistar and GK rats. As expected, a marked increase in ROS production was observed in the GK rat liver and kidney, with the increase more pronounced in the liver (72%) compared to the kidney (26%). The exercise regimen resulted in almost a 50% decrease in ROS production in the liver compared to a modest reduction observed in the kidney (Figure 1A). Similarly, a significant increase in NOX activity was also observed in the diabetic rat liver (almost 2-fold) and the kidney (70%), which decreased significantly (25%–50%) in the GK rat liver and kidney after exercise (Figure 1B). A significant increase (∼25%) in microsomal lipid peroxidation was observed in both liver and kidney of diabetic rats (Figure 1C). Exercise training, however, did not cause any significant changes in lipid peroxidation. On the other hand, protein carbonylation which increased significantly in GK rat liver and kidney (60%–70%) was brought down to almost control values after exercise (Figure 1D).

### Modulation of antioxidant levels in GK rat liver and kidney after exercise

**FIGURE 2:**
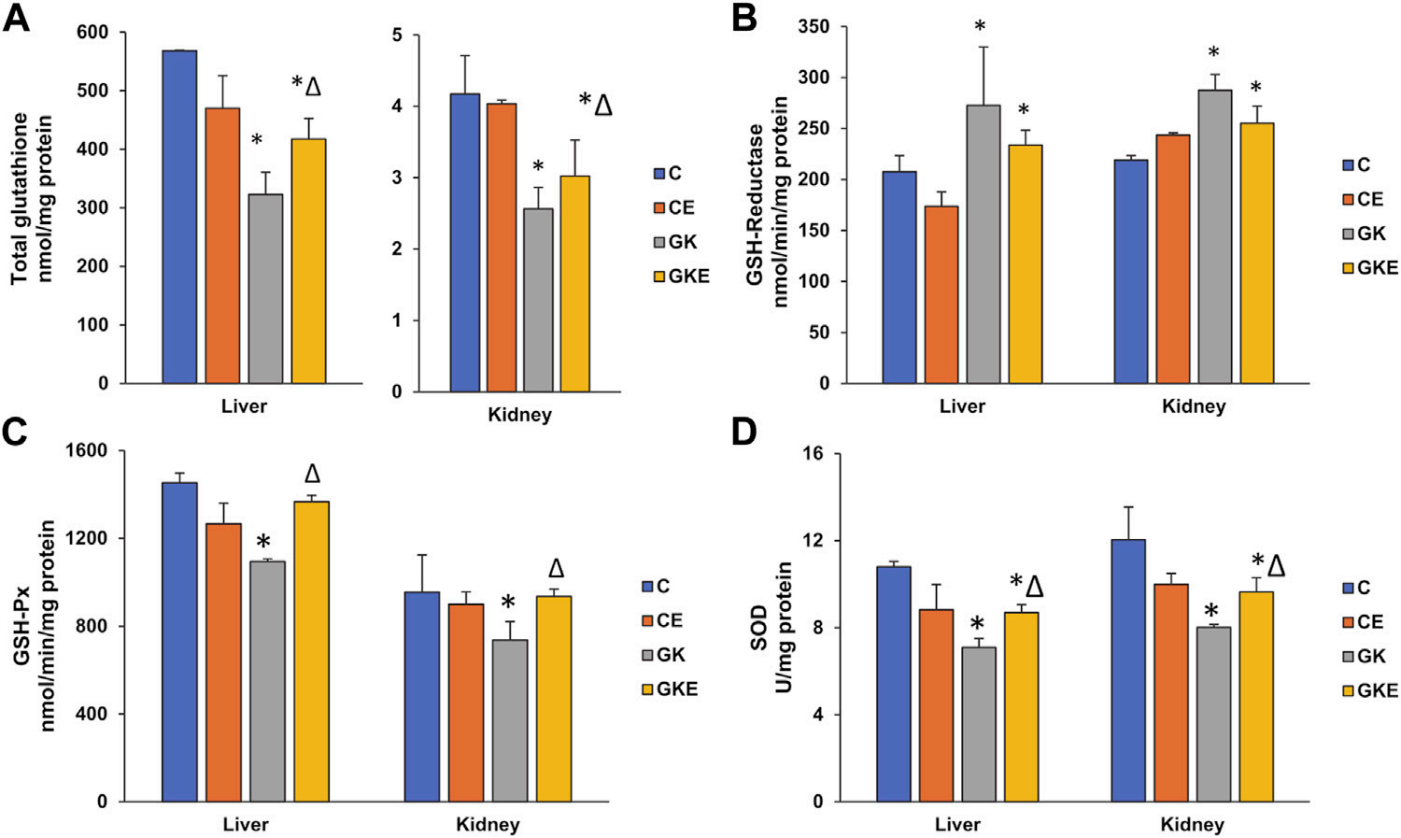
Exercise-induced modulation of antioxidant levels in the liver and kidney of control Wistar (C) and diabetic GK (GK) and exercise-trained control (CE) and GK (GKE) rats (*n* = 5). Enzymatic and non-enzymatic antioxidant levels were analyzed in the different sub-groups of control and GK rat tissues. Total GSH levels **(A)** were measured in the rat tissues as the enzymatic conversion of oxidized glutathione to reduced glutathione using DTNB as substrate. Glutathione reductase **(B)** and GSH-Px **(C)** activities were measured using GSSG/NADPH and cumene hydroperoxide as substrates respectively and SOD **(D)** activity was measured using the SOD assay kit as described in the Materials and Methods. Histogram values are mean ± of three independent experiments and statistical significance is shown as asterisks, which was fixed as *p* < 0.05 (* indicates statistical significance compared with control Wistar rat tissues while ∆ indicates statistical significance compared with diabetic GK rat tissues).

The level of GSH, an endogenous antioxidant synthesized abundantly in the liver was significantly reduced in both the liver and kidney (50%–60%) of GK diabetic rats (Figure 2A). Exercise significantly increased the GSH levels (20%–30%) in both tissues. The activity of GSH-reductase, which recycles oxidized glutathione to its reduced form, was significantly increased (30%) in both tissues (Figure 2B). Exercise caused a mild decrease (12%–15%) in the activity, which could be the reason for the still reduced levels of glutathione after exercise in both the diabetic tissues. The activity of the GSH-Px enzyme was reduced (about 25%) in both the liver and kidney of GK rats (Figure 2C). Exercise caused almost a 25% increase in activity in the GK rat tissues. Superoxide dismutase also showed a significant decrease (almost 35%) in enzyme activity in the liver and kidney of GK rats, which recovered moderately (20%–25%) after exercise (Figure 2D).

### Exercise-induced alterations in CYP 450 enzyme activities in GK rat liver and kidney

**FIGURE 3:**
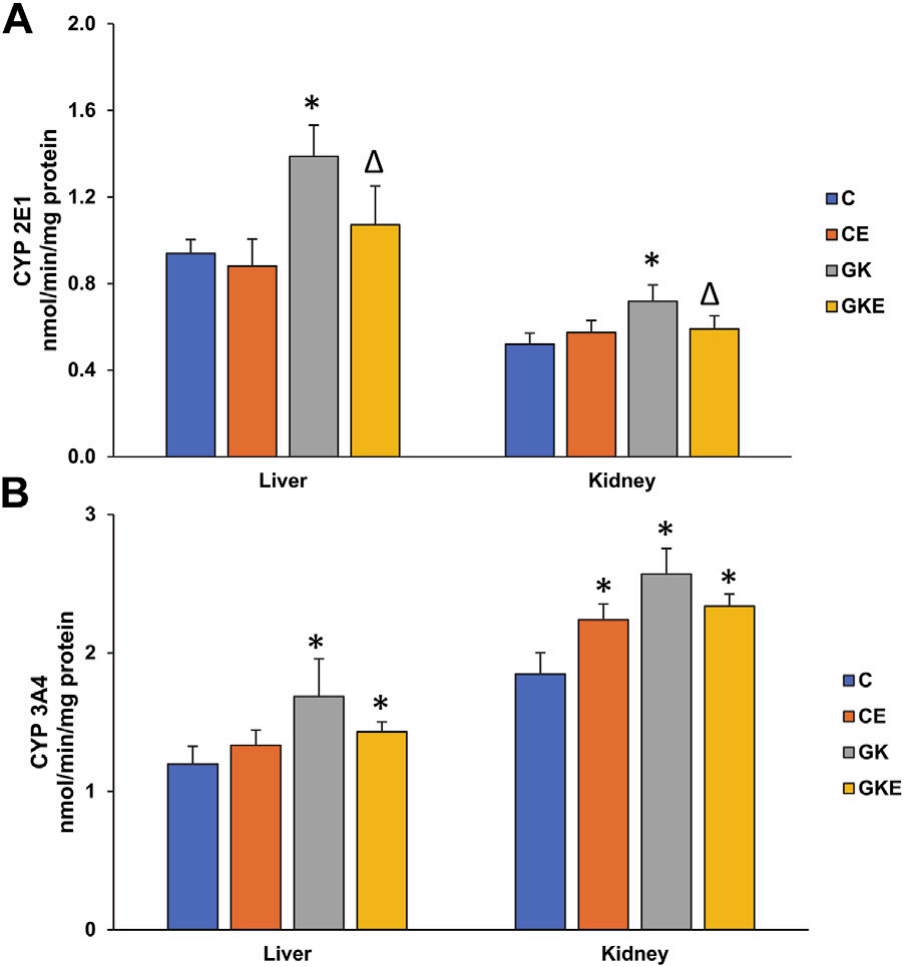
Exercise-induced alterations in CYP 450 enzyme activities in the liver and kidney of control Wistar (C) and diabetic GK (GK) and exercise-trained control (CE) and GK (GKE) rats (*n* = 5). Microsomal CYP 2E1 **(A)** and CYP 3A4 **(B)** were measured using dimethyl nitrosamine and erythromycin as substrates as described in the Materials and Methods. Histogram values are mean ± of three independent experiments and statistical significance is shown as asterisks, which was fixed as *p* < 0.05 (* indicates statistical significance compared with control Wistar rat tissues while ∆ indicates statistical significance compared with diabetic GK rat tissues).

As seen in our previous studies, CYP 450 2E1 and 3A4 enzymes were activated in diabetic rat liver and kidney tissues (Figure 3). A significant increase (40%–50%) in CYP 450 2E1 activity was observed in the diabetic rat tissues, which was alleviated by exercise training (Figure 3A). Similarly, CYP 450 3A4 activity also showed a significant (40%) increase in the GK rat liver and kidney tissues (Figure 3B). Exercise training, however, did not cause any significant decrease in enzyme activity. Exercise training, on the other hand, also caused a moderate increase in enzyme activity in the control Wistar rat kidney.

### Exercise-induced alterations in energy metabolizing enzyme activities in GK rat liver and kidney

**FIGURE 4:**
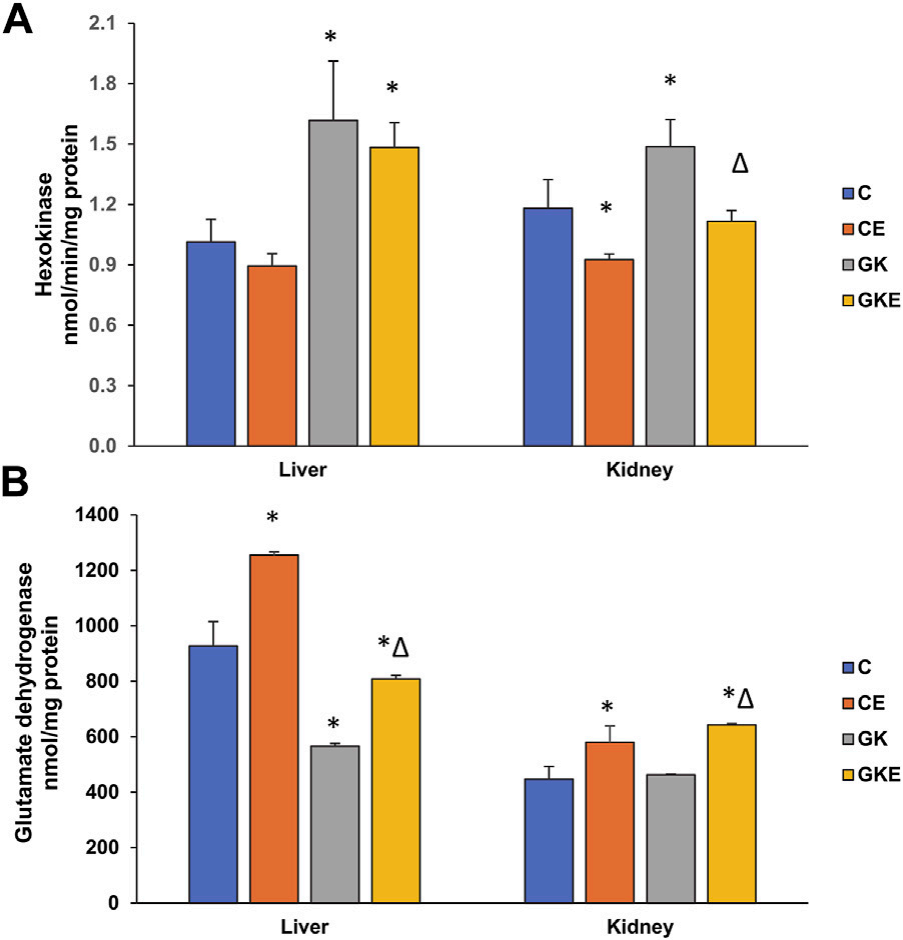
Exercise-induced alterations in energy metabolizing enzyme activities in the liver and kidney of control Wistar (C) and diabetic GK (GK) and exercise-trained control (CE) and GK (GKE) rats (*n* = 5). Hexokinase activity **(A)** was measured as the NADH generated using the hexokinase assay kit and glutamate dehydrogenase **(B)** was measured using a coupled enzyme assay kit as per the manufacturer’s instructions. Histogram values are mean ± of three independent experiments and statistical significance is shown as asterisks, which was fixed as *p* < 0.05 (* indicates statistical significance compared with control Wistar rat tissues while ∆ indicates statistical significance compared with diabetic GK rat tissues).

To study the alterations in energy metabolism, we investigated the glucose phosphorylating enzyme, hexokinase, in the liver and kidney of control and GK rats with and without exercise. We observed a significant increase (60%) in hexokinase activity in the GK rat liver, which remained higher than control levels even after exercise (Figure 4A). On the other hand, an increase (25%) in hexokinase activity was observed in the GK rat kidneys, which recovered significantly after exercise.

On the other hand, the activity of glutamate dehydrogenase (GDH), a mitochondrial Krebs’ cycle-associated enzyme, was decreased significantly in the GK rat liver (40%), which recovered after the exercise regimen (Figure 4B). The control Wistar rat liver also showed a significant increase in enzyme activity after exercise. However, the GK rat kidneys did not show a significant change in activity, though increased activities were observed after exercise in both the control Wistar and GK rats. This shows the differential responses of exercise training in different tissues presumably associated with their energy needs and metabolism.

### Effects of exercise on mitochondrial bioenergetics in GK rat liver and kidney

**FIGURE 5:**
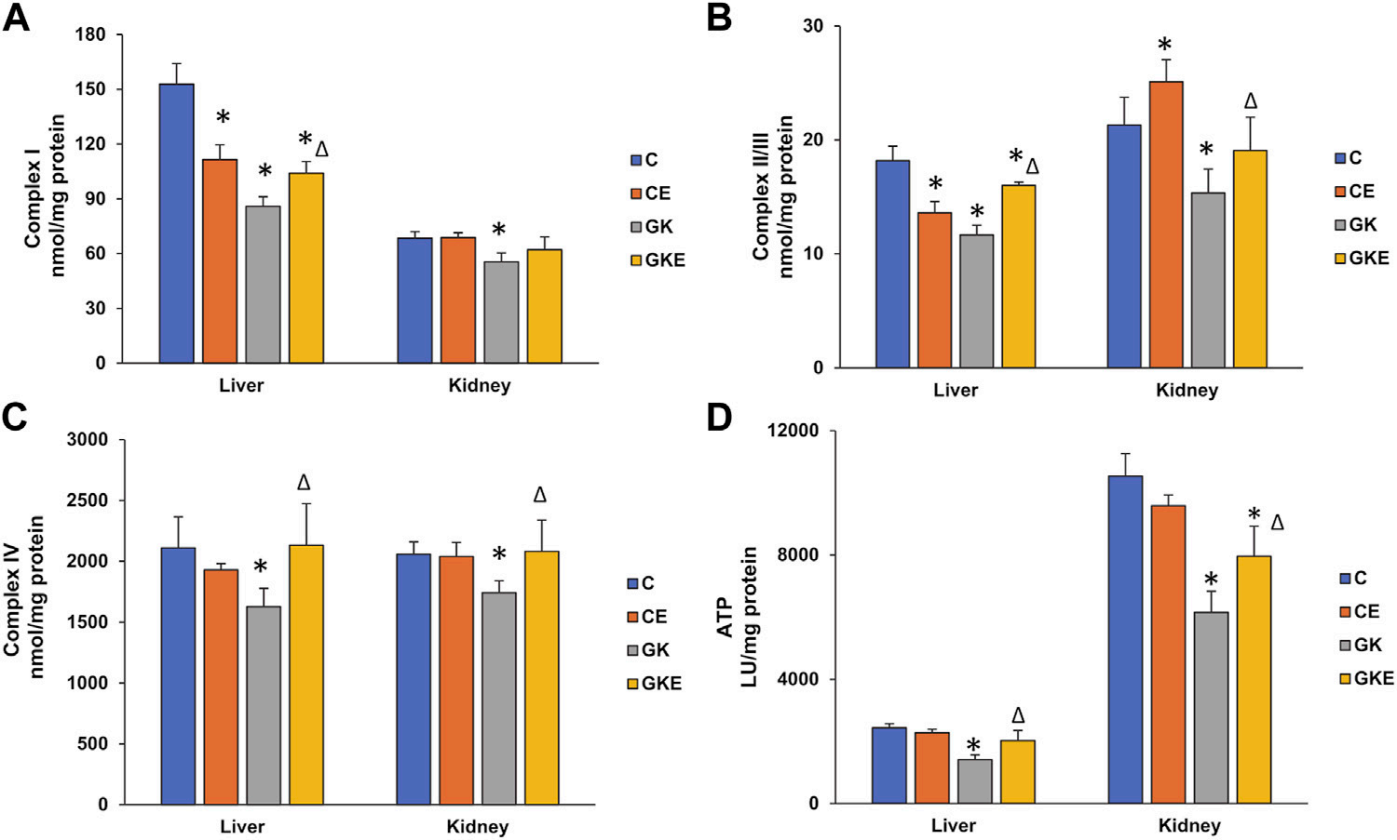
Exercise-induced alterations in mitochondrial bioenergetics. Activities of mitochondrial respiratory complexes from freshly isolated mitochondria were measured in the liver and kidney of control Wistar (C) and diabetic GK (GK) and exercise-trained control (CE) and GK (GKE) rats (*n* = 5). Activities of Complex I **(A)**, Complex II/III **(B)**, and Complex IV **(C)** were measured by the methods of Birch-Machin and Turnbull using coenzyme Q2, succinate, and reduced cytochrome c as substrates as described in the Materials and Methods. The total ATP levels **(D)** were measured using the ATP somatic cell assay kit (Sigma-Aldrich Fine Chemicals, St Louis, MO, United States) and the luminescence was read immediately using the TD-20/20 luminometer. Histogram values are mean ± of three independent experiments and statistical significance is shown as asterisks, which was fixed as *p* < 0.05 (* indicates statistical significance compared with control Wistar rat tissues while ∆ indicates statistical significance compared with diabetic GK rat tissues).

To further assess the effects of exercise on mitochondrial bioenergetics, we studied the activities of mitochondrial respiratory complexes in non-exercised and exercised control Wistar and GK diabetic rat liver and kidney. A marked reduction in respiratory complex I, II/III, and IV activities were observed in both liver and kidney of GK rats (Figures 5A–C). A significant increase in the activities of the mitochondrial complexes was observed after exercise in these tissues. However, a slight decrease in activities of complex I and II/III were observed in the control rat liver after exercise. This could be due to the increased demands and utilization of substrate causing a slight increase in ROS production after exercise. An increase in complex II/III activity was observed after exercise in the control Wistar rat kidney. Similarly, ATP levels were also ameliorated in both the tissues of GK rats, which improved after the exercise regimen (Figure 5D). No significant changes in ATP levels were observed in the exercised control Wistar rat tissues. These results signify the beneficial effects of exercise on enhancing energy metabolism in diabetic animals.

### Exercise-induced alterations in the expression of oxidative stress marker proteins in GK rat liver and kidney

**FIGURE 6:**
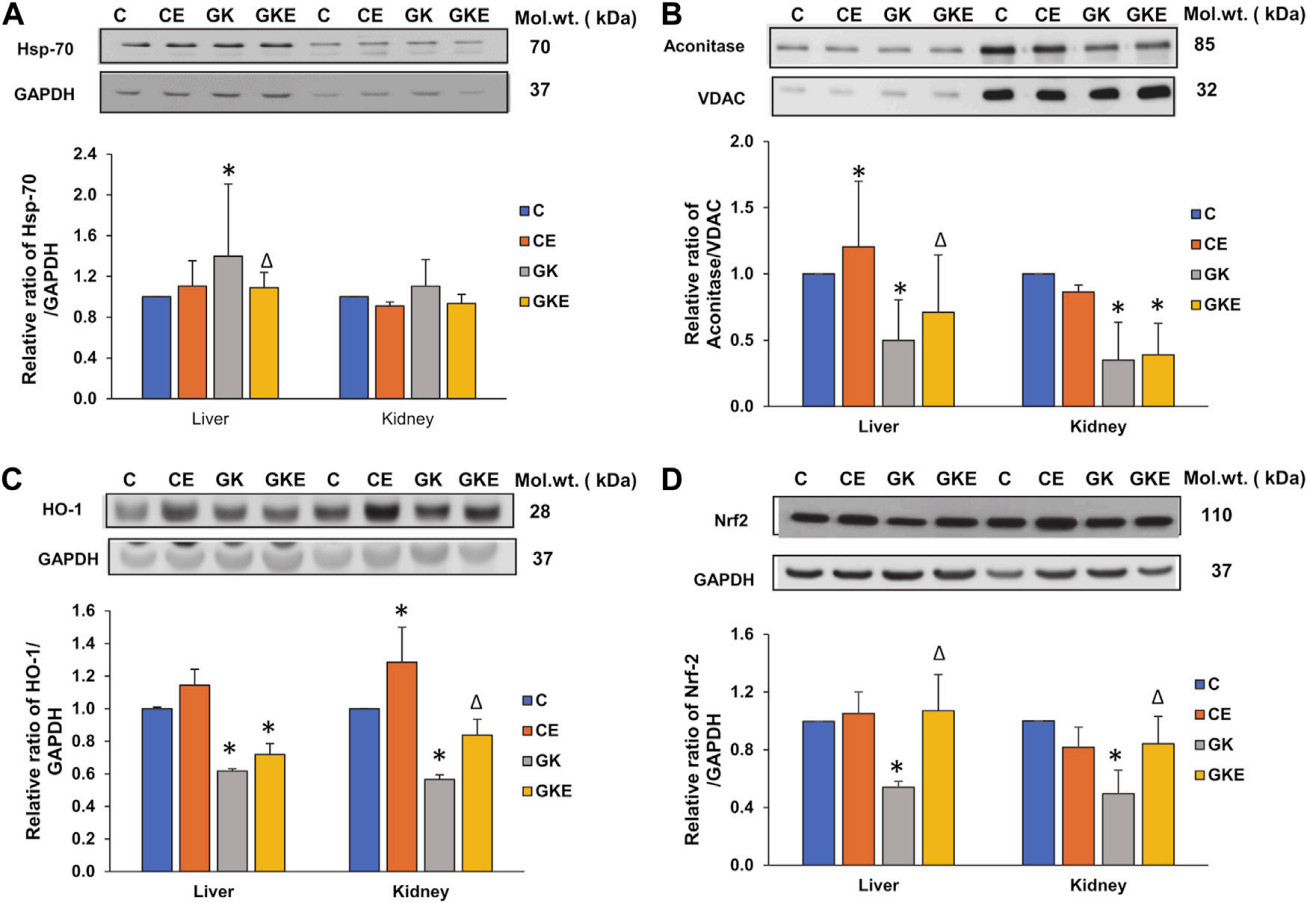
Exercise-induced alterations in the expression of oxidative stress marker proteins in the liver and kidney of control Wistar (C) and diabetic GK (GK) and exercise-trained control (CE) and GK (GKE) rats (*n* = 5). Cellular fractions were electrophoretically separated by 7.5%–12% SDS-PAGE, transferred onto nitrocellulose membranes, and immunoblotted with stress-sensitive marker proteins, Hsp-70 **(A)**, mitochondrial Aconitase **(B)**, HO-1 **(C)** and Nrf2 **(D)** and visualized using the Sapphire Biomolecular Imager (Azure Biosystems, Dublin, OH, United States) or by exposure to X-ray films. GAPDH and VDAC were used as loading controls for cytosolic and mitochondrial fractions respectively. Immunoreactive bands were quantitated using Image Studio Lite, ver.5.2 (LI-COR Biosciences, Lincoln, NE, United States). Histograms represent the relative ratio of protein over their respective loading controls from three independent experiments. Statistical significance is shown as asterisks, which was fixed as *p* < 0.05 (* indicates statistical significance compared with control Wistar rat tissues while ∆ indicates statistical significance compared with diabetic GK rat tissues). Molecular weights of the respective proteins are indicated in kDa. Original western blot figures are shown in the Supplementary Figure S6.

To confirm the defensive effects of exercise on oxidative stress in diabetes, we investigated the expression of stress-sensitive marker proteins in the liver and kidney of control Wistar and GK rats with or without exercise (Figure 6). A significant increase in the expression of stress-inducible Hsp-70 protein was observed in the liver (40%) of GK rats which came close to control values after exercise (Figure 6A). The kidney of GK rats, however, showed only a mild increase in expression, which again came back to control values after exercise. This confirms the differential response of tissues to stress. Another mitochondrial ROS-sensitive marker, Aconitase was significantly decreased (50%–60%) in both the tissues of GK rats (Figure 6B). The exercise regimen again enhanced the depleted enzyme expression in the liver of GK rats. Exercise did not cause a significant change in the GK rat kidneys. Next, we assessed the expression of antioxidative defense responsive marker proteins, HO-1 and Nrf2 (Figures 6C,D). The expression of both these marker proteins was significantly reduced in the liver and kidney of GK rats. However, significant recovery (30%–50%) was observed with the Nrf2 protein in both tissues of GK rats. This suggests a partial recovery after exercise.

### Exercise-induced alterations in the expression of inflammatory markers in GK rat liver and kidney

**FIGURE 7:**
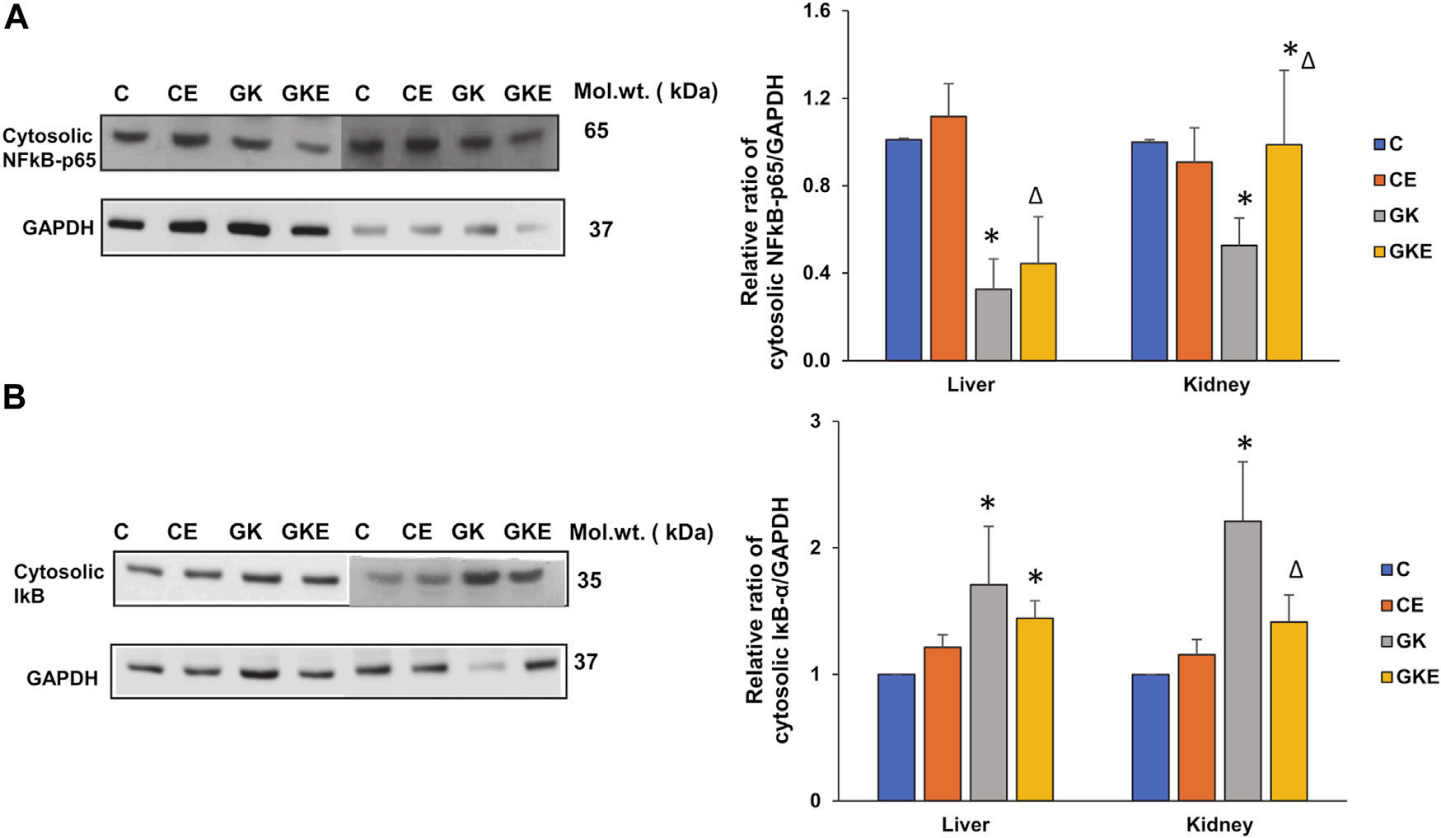
Exercise-induced alterations in the expression of inflammatory marker proteins in the liver and kidney of control Wistar (C) and diabetic GK (GK) and exercise-trained control (CE) and GK (GKE) rats (*n* = 5). Cytosolic fractions were electrophoretically separated by 10% SDS-PAGE, transferred onto nitrocellulose membranes and immunoblotted with inflammatory marker proteins, NFκB-p65 **(A)**, and IκB **(B)** and visualized using the Sapphire Biomolecular Imager (Azure Biosystems, Dublin, OH, United States) or by exposure to X-ray films. GAPDH was used as the loading control. Immunoreactive bands were quantitated using Image Studio Lite, ver.5.2 (LI-COR Biosciences, Lincoln, NE, United States). Histograms represent the relative ratio of protein to the loading control from three independent experiments. Statistical significance is shown as asterisks, which was fixed as *p* < 0.05 (* indicates statistical significance compared with control Wistar rat tissues while ∆ indicates statistical significance compared with diabetic GK rat tissues). Molecular weights of the respective proteins are indicated in kDa. Original western blot figures are shown in Supplementary Figure S7.

In addition to oxidative stress, inflammation is a hallmark of diabetes. We, therefore, investigated the expression of inflammatory markers NFkB-p65 and IκB. A decrease in the expression of NFkB-p65 and an increase in the expression of IκB were observed in the cytosolic fraction with significant recovery after the exercise regimen in both the tissues of GK rats (Figures 7A,B). This confirms increased inflammation in the diabetic tissues.

### Exercise-induced alterations in insulin signaling in GK rat liver and kidney

**FIGURE 8:**
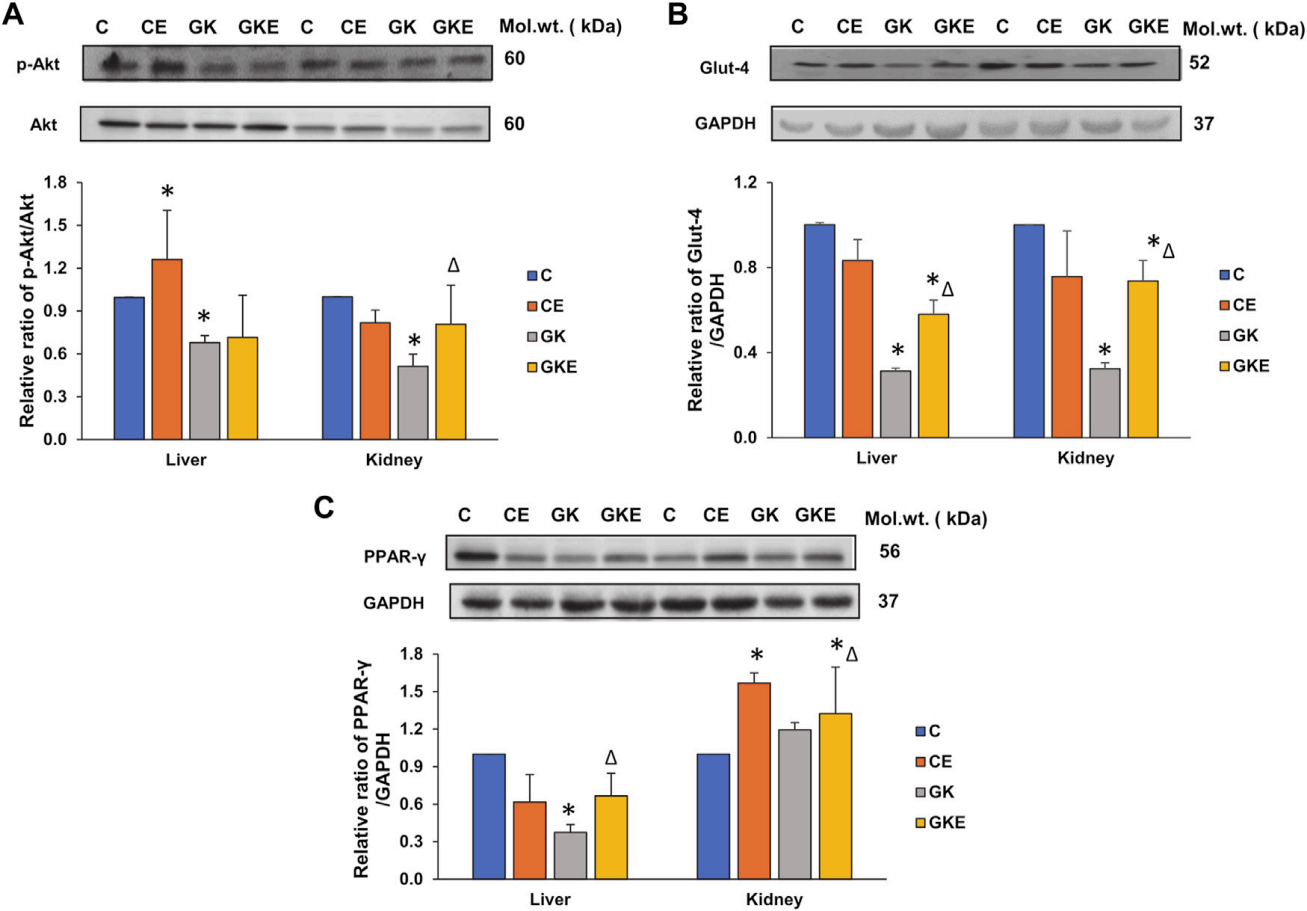
Exercise-induced alterations in the expression of cell-signaling metabolic marker proteins in the liver and kidney of control Wistar (C) and diabetic GK (GK) and exercise-trained control (CE) and GK (GKE) rats (*n* = 5). Cytosolic fractions were electrophoretically separated by 12% SDS-PAGE, transferred onto nitrocellulose membranes, and immunoblotted with cell-signaling marker proteins, p-Akt **(A)**, glucose transporter protein, Glut-4 **(B)**, and PPAR-γ **(C)** and visualized using the Sapphire Biomolecular Imager (Azure Biosystems, Dublin, OH, United States) or by exposure to X-ray films. Total protein or GAPDH was used as the loading control. Immunoreactive bands were quantitated using Image Studio Lite, ver.5.2 (LI-COR Biosciences, Lincoln, NE, United States). Histograms represent the relative ratio of protein to the loading control from three independent experiments. Statistical significance is shown as asterisks, which was fixed as *p* < 0.05 (* indicates statistical significance compared with control Wistar rat tissues while ∆ indicates statistical significance compared with diabetic GK rat tissues). Molecular weights of the respective proteins are indicated in kDa. Original western blot figures are shown in Supplementary Figure S8.

Inflammation in diabetes and its related metabolic disorders are known to cause disturbances in insulin signaling. Protein kinase B (Akt), a physiological regulator of cell growth and survival, also plays an important role in downstream insulin signaling and glucose uptake. To confirm the effects on insulin signaling, we checked the effect of exercise on the activation of Akt and expression of glucose transporter type 4 (Glut-4) in the liver and kidneys of GK rats. We observed a significant decline in the activation of Akt in both tissues (Figure 8A). However, exercise caused significant activation (58%) of Akt protein only in the kidney of GK rats. No appreciable change was observed in the liver of these animals. On the other hand, we observed an increase in the depleted expression of Glut-4 protein in both tissues of GK rats, indicating increased glucose uptake (Figure 8B). Again, the increase was more profound in the kidney compared to the liver, suggesting tissue-specific differences in insulin signaling and glucose uptake.

To further confirm the increased insulin sensitivity after exercise, we checked the expression of PPAR-γ, a major regulator of glucose and fatty acid metabolism. We observed a significant decrease (60%) decrease in the expression of PPAR-γ in the liver of GK rats, which increased significantly after exercise. No significant change was observed in the control liver after exercise. Kidney, on the other hand, showed increased PPAR-γ in both the control and GK rats after exercise. No appreciable change was observed in the kidney of GK rats. This again confirms the differential responses of the tissues to insulin signaling based on their demands for energy metabolism.

### Exercise-induced alterations in the expression of energy regulators in GK rat liver and kidney

**FIGURE 9:**
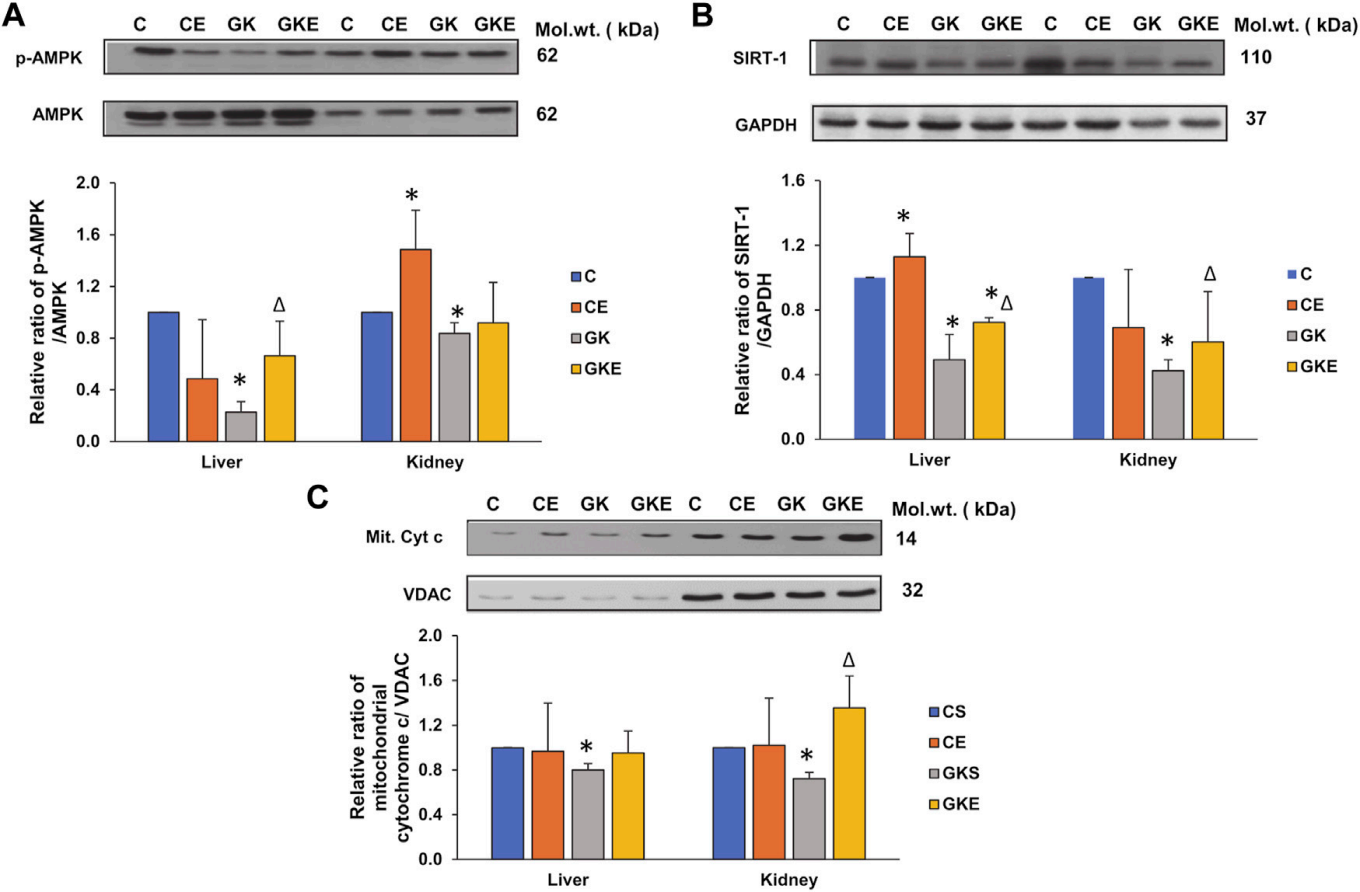
Exercise-induced alterations in the expression of energy regulator marker proteins in the liver and kidney of control Wistar (C) and diabetic GK (GK) and exercise-trained control (CE) and GK (GKE) rats (*n* = 5). Cytosolic and mitochondrial fractions were electrophoretically separated by 7.5%–12% SDS-PAGE, transferred onto nitrocellulose membranes, and immunoblotted with proteins regulating energy metabolism, p-AMPK **(A)**, SIRT-1 **(B)**, and mitochondrial cytochrome c **(C)** and visualized using the Sapphire Biomolecular Imager (Azure Biosystems, Dublin, OH, United States) or by exposure to X-ray films. Total protein and GAPDH were used as loading controls for p-AMPK and SIRT-1 respectively, and VDAC for the mitochondrial fraction. Immunoreactive bands were quantitated using Image Studio Lite, ver.5.2 (LI-COR Biosciences, Lincoln, NE, United States). Histograms represent the relative ratio of protein over their respective loading controls from three independent experiments. Statistical significance is shown as asterisks, which was fixed as *p* < 0.05 (* indicates statistical significance compared with control Wistar rat tissues while ∆ indicates statistical significance compared with diabetic GK rat tissues). Molecular weights of the respective proteins are indicated in kDa. Original western blot figures are shown in Supplementary Figure S9.

AMPK and SIRT-1 are key regulators of energy metabolism, controlling cellular energy homeostasis. We investigated the expression of these energy regulators in the liver and kidney of control Wistar and GK diabetic rats with or without exercise. We observed a significant decline in the AMPK phosphorylation in the liver and kidney of GK rats, which increased significantly in the liver after exercise (Figure 9A). Kidney, however, showed a mild increase in phosphorylation in GK rats but significant activation was observed in control Wistar rats after exercise. Similarly, increased expression of SIRT-1 protein was observed after exercise in the liver and kidney of GK animals, which showed a significant decrease in protein expression (Figure 9B). Since SIRT-1 has also been known to play a critical role in regulating mitochondrial function and biogenesis, we checked the mitochondrial cytochrome c levels in the liver and kidney of control Wistar and GK rat liver and kidney. We observed a mild decrease (about 20%) in the cytochrome c level in the liver and a significant decrease (30%) in the kidney of GK rats (Figure 9C). Exercise improved cytochrome c levels significantly (80%) in the kidney of GK rats, while the liver showed only a mild increase in expression after exercise.

## Discussion

Hyperglycemia-induced chronic inflammation and metabolic dysregulation play a crucial role in the micro- and macro-vascular complications of diabetes and are associated with long-term dysfunction of various organs, especially the liver and kidneys (Gul et al., 2002; Lima et al., 2015; Amaral et al., 2018; Liu et al., 2019). Through hyperglycemia, hyperlipidemia, and associated complications, diabetes induces oxidative stress that can result in mitochondrial dysfunction, insulin resistance as well as lipid, protein, and DNA breakdown and cause damage to multiple organs (Gul et al., 2002; Ghosh et al., 2009; Qi et al., 2011). Due to their non-obese, mild hyperglycemic, and hyperlipidemic characteristics, the GK rats have been widely used as an experimental model for type 2 insulin-resistant diabetes (Guest, 2019).

Despite the benefits of pharmacological therapies, prophylactic approaches such as physical exercise, besides caloric restriction are considered the cornerstone in the management of type 2 diabetes (Ghosh et al., 2009; Amaral et al., 2018; Foudi and Legeay, 2021). Regular exercise has long been considered to be an effective non-pharmacological approach to strengthen anti-oxidant defenses and improve mitochondrial function, insulin sensitivity, and metabolic control in several tissues and metabolic disorders (Qi et al., 2011; Lima et al., 2015; Amaral et al., 2018). Although numerous studies have demonstrated the multiple beneficial effects of exercise, a clear understanding of the molecular basis for redox and metabolic homeostasis is still inchoate (Amaral et al., 2018; Farzanegi et al., 2019; Foudi and Legeay, 2021). We, therefore, aimed to elucidate the effects of moderate, regular exercise on the molecular basis of oxidative stress, antioxidant responses, mitochondrial functions, and metabolic homeostasis in the liver and kidneys of GK diabetic rats.

Our previous study in GK rat pancreas demonstrated exercise-induced beneficial effects by improving energy metabolism, antioxidant responses and mitochondrial functions, and metabolic adaptation (Raza et al., 2016). Our current study on the same cohort of animals demonstrated the beneficial effects of moderate exercise in reducing oxidative stress and inflammation, improving insulin signaling, antioxidant responses, and redox homeostasis, and enhancing mitochondrial bioenergetics and energy metabolism presumably *via* the AMPK/SIRT-1/PPAR-γ signaling pathways.

Oxidative stress induced by mitochondrial and non-mitochondrial ROS and inflammation has been reported as the mechanism underlying insulin resistance (Qi et al., 2011), cell death, and tissue injury (Ghosh et al., 2009; Qi et al., 2011; Golbidi et al., 2012; Amaral et al., 2018; Farzanegi et al., 2019; Monno et al., 2021). Our results showed an increase in ROS production and activation of non-mitochondrial NADPH oxidase (NOX), accompanied by increased lipid peroxidation and protein carbonylation in the liver and kidney of GK rats. Exercise-induced reduction in ROS production is presumably due to the significant reduction in the NOX activities in these tissues. Though no significant change was observed in lipid peroxidation in the liver and kidneys after exercise, a significant reduction in protein peroxidative carbonylation was observed in both tissues. Regular moderate exercise did not show any significant change in the tissue of control Wistar rats, suggesting minimal oxidative stress in the control animals.

Excessive ROS production is known to suppress antioxidant defense systems and physical exercise training has been shown to upregulate antioxidant defense mechanisms in several tissues and metabolic disorders (Golbidi et al., 2012; Lima et al., 2015). Glutathione, an abundant endogenous antioxidant has been reported to be decreased in type 2 diabetic patients (Sekhar et al., 2011; Lagman et al., 2015; Gutiérrez-Camacho et al., 2020). In our study, we observed a decrease in the GSH pool in the GK rat tissues, which increased significantly with exercise. Consequently, we also observed an increase in glutathione reductase activity in the GK rats, which could be a compensatory mechanism to recycle the reduced glutathione. Decreased GSH levels have been reported in the skeletal muscle of GK diabetic rats (Qi et al., 2011). However, there are also reports of no appreciable change in renal glutathione levels in streptozotocin-induced diabetic rats with or without exercise (Amaral et al., 2018). This could be because the imbalance of the redox state in this diabetic model was not sufficient to reduce the antioxidant defenses. Our study further demonstrated a decrease in the activities of the enzymatic antioxidants, glutathione peroxidase, and superoxide dismutase in the liver and kidney of diabetic rats. Decreased levels of cytosolic SOD have been reported in diabetic liver and kidneys, which improved with exercise (Ghosh et al., 2009; Lima et al., 2015). This was confirmed in our study, where exercise was able to significantly improve the depleted SOD levels in the GK rat tissues. Treadmill exercise training has also been shown to increase hepatic glutathione peroxidase levels in STZ-diabetic rats (Lima et al., 2015). This was again confirmed in our study. Exercise training augmented the reduced peroxidase activity in the kidney and liver of GK rats.

The drug metabolizing enzymes, cytochrome P450 2E1 and 3A4 were also found to be activated in the liver and kidney of diabetic GK rats. This was in confirmation of our earlier studies on different diabetic rat models (Raza et al., 2004; Raza et al., 2012; Raza et al., 2013; Raza et al., 2015; Raza et al., 2016). Exercise alleviated the activities of the CYP 450 enzymes significantly in the liver of GK rats. The kidney, however, showed only a modest decrease in activities after exercise. This could be because the liver plays a central role in the detoxification of exogenous and endogenous material.

At the center of the cellular response to oxidative stress is the keap 1-Nrf2-antioxidant response elements (ARE) pathway, which helps in the maintenance of cellular homeostasis and detoxification by regulating the expression of many antioxidant proteins (Tu et al., 2019). Nrf2 plays a central role in the regulation of cellular defense mechanisms. Under oxidative stress conditions, it can activate a series of antioxidative and various cytoprotective proteins including heme oxygenase-1 (HO-1), glutathione peroxidase, and SOD. In our study, we observed a decrease in the expression of Nrf2 and HO-1proteins in the liver and kidney of GK rats, which were activated after exercising. Nrf2 depletion has been shown to cause renal oxidative and nitrosative stress in hyperglycemia-induced STZ mice (Yoh et al., 2008).

In addition to oxidative stress, chronic inflammation has been the hallmark of diabetes and its related complications. Nrf2, HO-1 and NFκB pathways are known to regulate cellular redox homeostasis (Bellezza et al., 2012, 2018). Studies have shown that stimulation of NFκB induces Nrf2, which upregulates HO-1 expression, which in turn, terminates the NFκB activation. (Bellezza et al., 2012). This could be the reason for the activation of Nrf2 and HO-1 and alleviation of NFκB activation that we observed in our study after exercise training in GK rat tissues. As per the canonical signaling pathway, intra- or extracellular stimuli such as ROS leads to IκB phosphorylation and degradation, which leads to the activation of NFκB (Patel and Santani, 2009). Our study also demonstrated a decreased level of cytosolic NFκB and an increased expression of IκB in the liver and kidney of GK rats, indicating increased nuclear translocation of NFκB, which recovered significantly after exercising.

Sirtuin 1 (SIRT-1), a cellular energy sensor and major regulator of energy metabolism, integrates cellular metabolism and inflammation by regulating downstream signaling pathways (Liu et al., 2019). Researchers have established a direct and antagonistic relationship between the SIRT-1 and NFκB, in which SIRT-1 can deacetylate the p65 subunit of NFκB, triggering its transport from the nucleus to the cytoplasm (de Gregorio et al., 2020). Also, loss of SIRT-1 has been shown to acetylate NFκB, thereby impairing mitochondrial biogenesis and triggering inflammation in type 2 diabetic fatty Wistar rats (Kitada et al., 2011), which was seen to be partially normalized by moderate exercise training in diabetic fatty mice (Liu et al., 2019). Our study also showed exercise-induced increased expression of cytosolic NFκB-p65 associated with upregulation of SIRT-1 in the liver and kidney of diabetic GK rats. Increased expression of SIRT-1 was also seen in the liver of control rats after exercise. This again points to the liver being proactive in the detoxification of toxicant material.

Studies, including our own, have shown mitochondrial dysfunction, including depressed mitochondrial respiratory complex activities and ultra-structural abnormalities in type 1 and type 2 diabetic rat models (Raza et al., 2016; Tang et al., 2018; Liu et al., 2019; John et al., 2022). Impaired mitochondrial capacity for glycogen and lipid oxidation has also been closely related to insulin resistance of peripheral tissues and also includes reduced oxygen consumption and ATP production and increased oxidative stress and attenuation after exercise training (Qi et al., 2011). Our present study also confirmed reduced activities of the mitochondrial respiratory complexes and depressed ATP production in diabetic rat liver and kidneys. In addition, we also observed a decreased expression of aconitase, a mitochondrial ROS-sensitive marker protein, and a decrease in the expression of mitochondrial cytochrome c in the diabetic tissues, indicating increased mitochondrial stress. Exercise caused significant recovery in the activities of the complexes and also increased ATP production in the liver and kidneys of diabetic rats. In addition, we also observed activation of the depressed glutamate dehydrogenase (GDH) activity in GK diabetic rat tissues after exercise training. GDH is also linked to redox homeostasis and cell signaling processes (Plaitakis et al., 2017). Our previous study also showed that increased GDH activity improved insulin sensitivity in the pancreas of diabetic GK rats after exercise training (Raza et al., 2016).

Another key energy sensor is AMP-activated protein kinase (AMPK), an insulin-independent regulator of glucose uptake, which on activation by exercise, regulates downstream targets involved in mitochondrial biogenesis, energy metabolism, and oxidative capacity (Ruderman et al., 2013; Tanaka et al., 2020). Our current study has also shown increased AMPK activity and improved mitochondrial function after regular moderate exercise in the liver and kidney of diabetic GK rats. Besides being a regulatory factor for energy status and glucose metabolism, it also plays a crucial role in regulating lipid metabolism, oxidative stress, and inflammatory responses (Green et al., 2022). Fang et al. have shown that these mitigative effects are achieved by activation of the Nrf2/keap1 pathway and inhibition of the NFκB pathway (Fang et al., 2022). Activation of AMPK and SIRT-1 has been shown to regulate hexokinase activity along with the regulation of glycolytic and mitochondrial energy metabolism (Cantó et al., 2009; Ren et al., 2022). Our present finding also shows a correlation between AMPK activation, increased SIRT-1 expression, and improved mitochondrial function in the liver and kidney of GK rats after exercising.

It is an indisputable fact that insulin regulation of glucose transport requires phosphorylation by hexokinases (Wasserman, 2022). In our recent study (John et al., 2022), we observed increased hexokinase activity in the liver and kidney of GK diabetic rats, confirming an increased adaptation by the tissues to phosphorylate glucose in response to mild hyperglycemia. Exercise training caused a significant decrease in activity in the kidney, though no significant change was observed in the liver. Activation of the PI3K/Akt/Glut-4 pathway has been known to be significantly involved in the regulation of insulin signaling and glucose metabolism and defects in the insulin signaling pathway, which lead to glucose homeostasis could lead to insulin resistance (Marinho et al., 2014; Rai et al., 2019; Kowalczuk et al., 2021; John et al., 2022). Our current study also showed activation of Akt and improved expression of Glut-4 protein after exercising, implying improved glucose homeostasis and increased insulin sensitivity. Increased Glut-4 expression is also correlated to increased activation of PPAR-γ, which improves insulin sensitivity through activation of the insulin signaling cascades and also facilitates improved energy metabolism (Kim and Ahn, 2004). Our present study also showed increased expression of PPAR-γ after exercise in the GK rat liver suggesting increased fatty acid oxidation.

## Conclusion

In summary, our study shows that exercise significantly improves redox homeostasis, glucose uptake, and energy metabolism, improves mitochondrial function, and possibly increases insulin sensitivity by activation of the AMPK/SIRT-1/PPAR-γ pathways. The main highlight of the present study is that we have demonstrated, for the first time, a correlation between exercise-induced alterations in the energy-sensing proteins, AMPK and SIRT-1, and the PPAR-γ dependent pathways, which regulate inflammatory responses and energy metabolism in diabetic rat tissues. This, in turn, ameliorates oxidative stress, mitochondrial dysfunction, and insulin resistance in the tissues of a non-obese model of type 2 diabetic GK rats.

## Data Availability

The original contributions presented in the study are included in the article/Supplementary Material, further inquiries can be directed to the corresponding author.
