# Supplementary material for: Exercise alleviates diabetic complications by inhibiting oxidative stress-mediated signaling cascade and mitochondrial metabolic stress in GK diabetic rat tissues
Source: Front Physiol. 2022 Dec 1;13:1052608. doi: 10.3389/fphys.2022.1052608 (PMC9751475; doi:10.3389/fphys.2022.1052608)
Supplement: Supplementary file 1 [file DataSheet1.PDF]

ORIGINAL WESTERN BLOT IMAGES FOR FIGURES 6-9

For Figure 6:

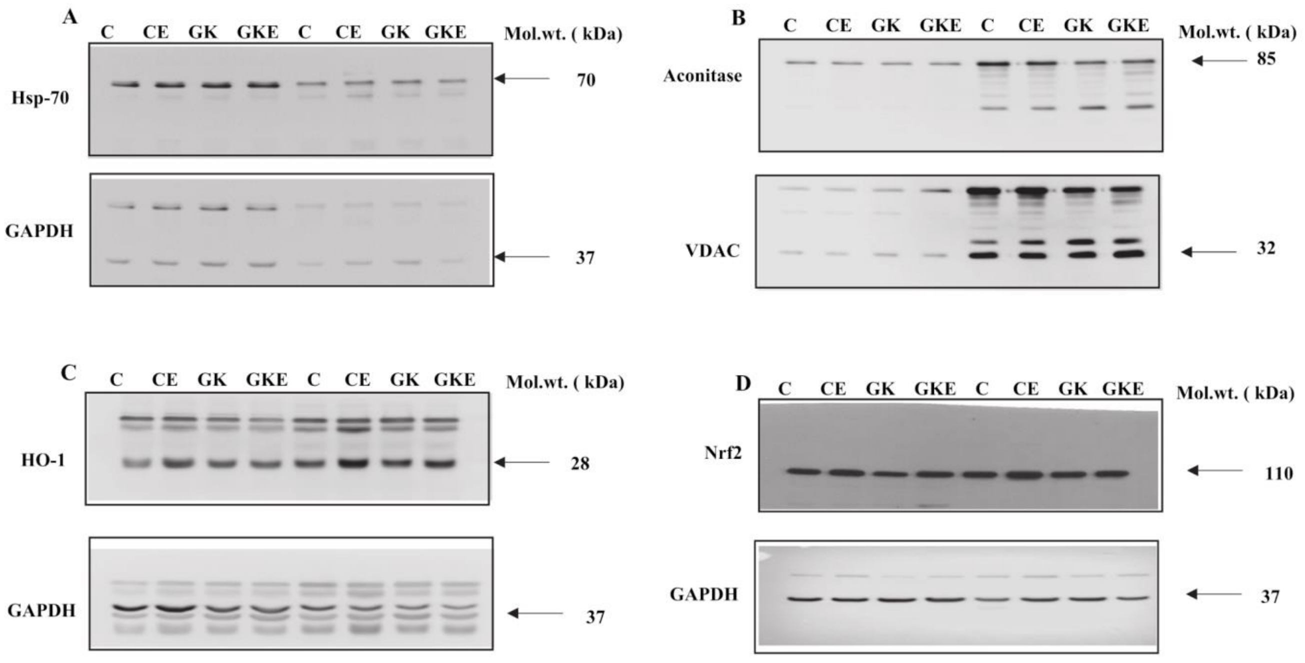

Supplementary Figure 6: Original blots of the stress-sensitive marker proteins, Hsp-70 (A), mitochondrial Aconitase (B), HO-1 (C), and Nrf2 (D) which were visualized using the Sapphire Biomolecular Imager (Azure Biosystems, Dublin, U.S.A) or by exposure to X-ray films. GAPDH and VDAC were used as loading controls for cytosolic and mitochondrial fractions respectively. Immunoreactive bands were quantitated using Image Studio Lite, ver.5.2 (LI-COR Biosciences, Lincoln, U.S.A). Molecular weights of the respective proteins are indicated in kDa.

For Figure 7:

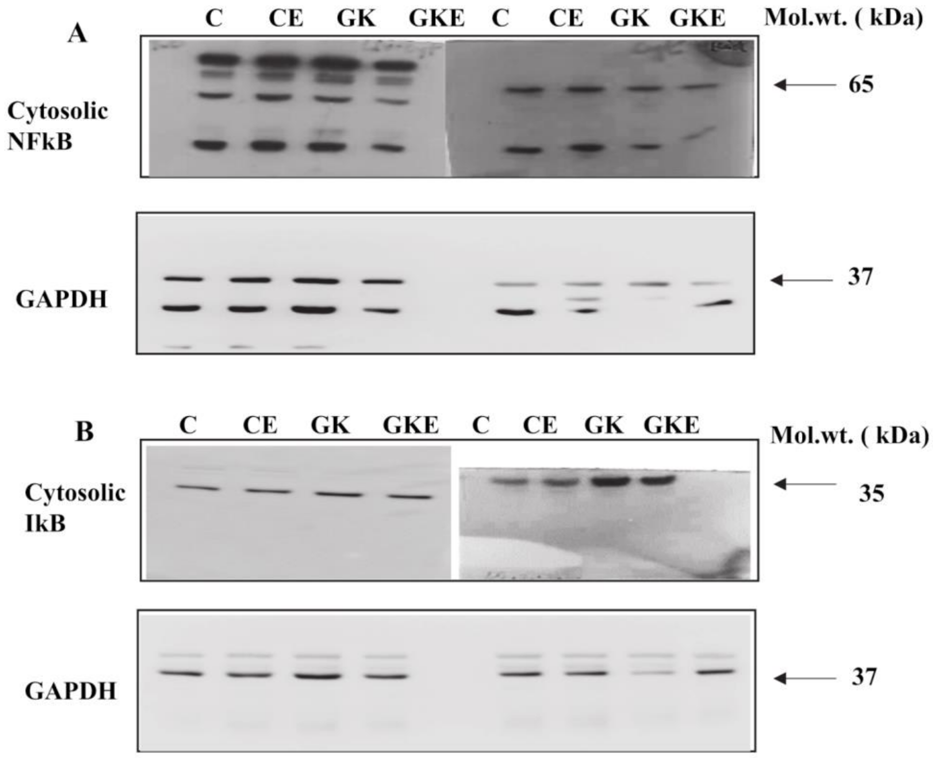

Supplementary Figure 7: Original blots of the inflammatory marker proteins, NFκB-p65 (A), and IκB (B)which were visualized using the Sapphire Biomolecular Imager (Azure Biosystems, Dublin, U.S.A) or by exposure to X-ray films. GAPDH was used as the loading control. Immunoreactive bands were quantitated using Image Studio Lite, ver.5.2 (LI-COR Biosciences, Lincoln, U.S.A). Molecular weights of the respective proteins are indicated in kDa.

For Figure 8:

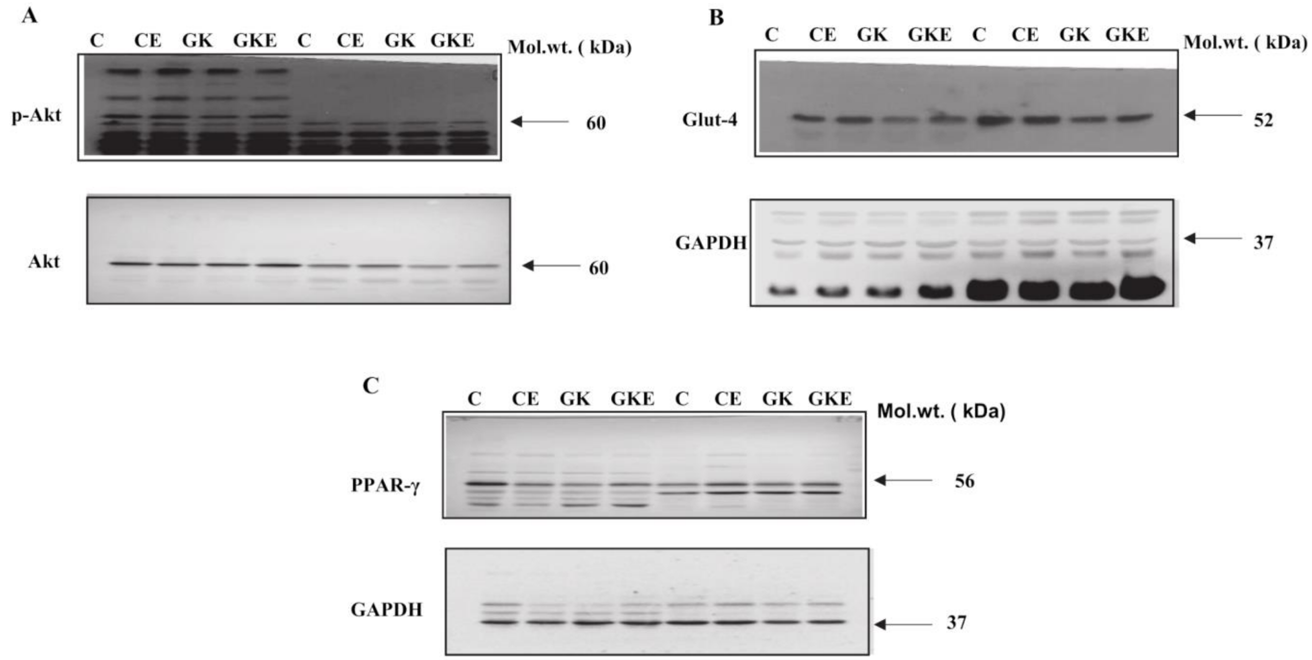

Supplementary Figure 8: Original blots of the cell-signaling marker proteins, p-Akt (A), glucose transporter protein, Glut-4 (B), and PPAR-γ (C) which were visualized using the Sapphire Biomolecular Imager (Azure Biosystems, Dublin, U.S.A) or by exposure to X-ray films. Total protein or GAPDH was used as the loading control. Immunoreactive bands were quantitated using Image Studio Lite, ver.5.2 (LI-COR Biosciences, Lincoln, U.S.A). Molecular weights of the respective proteins are indicated in kDa.

For Figure 9:

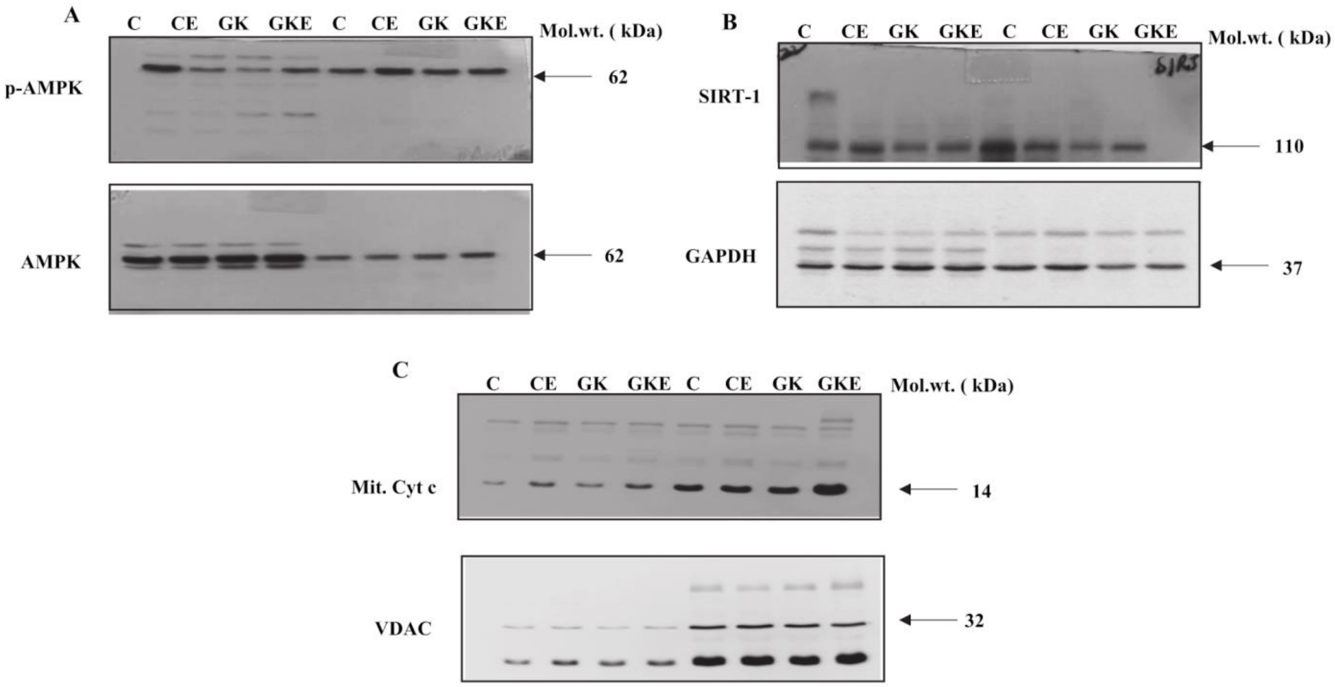

Supplementary Figure 9: Original blots of the proteins regulating energy metabolism, p-AMPK (A), SIRT-1 (B), and mitochondrial cytochrome c (C) which were visualized using the Sapphire Biomolecular Imager (Azure Biosystems, Dublin, U.S.A) or by exposure to X-ray films. Total protein and GAPDH were used as loading controls for p-AMPK and SIRT-1 respectively, and VDAC for the mitochondrial fraction. Immunoreactive bands were quantitated using Image Studio Lite, ver.5.2 (LI-COR Biosciences, Lincoln, U.S.A). Molecular weights of the respective proteins are indicated in kDa.
